# Assessment of health behaviors of primary school teachers based on their nutritional knowledge and physical activity: A cross-sectional study in the Asir Region

**DOI:** 10.1371/journal.pone.0318146

**Published:** 2025-01-27

**Authors:** Amani Alhazmi, Maha Ali, Adam Dawria, Bayapa Reddy Narapureddy, Manal Mohammed Hawash

**Affiliations:** 1 Department of Public Health, College of Applied Medical Sciences, King Khalid University, Abha, Saudi Arabia; 2 Department of Gerontological Nursing, Faculty of Nursing, Alexandria University, Egypt; King Abdulaziz University Faculty of Medicine, SAUDI ARABIA

## Abstract

**Background:**

Primary school teachers play a critical role as educators in imparting healthy eating behaviour and the importance of physical activity to prevent health issues. However, the teachers’ health behaviors have not been studied much, particularly in Saudi Arabia. Understanding these factors is essential to developing interventions that enhance teachers’ well-being and their ability to influence students positively. This study aims to assess nutritional knowledge and physical activity; to determine correlations between health behaviour factors and Body Mass Index (BMI), and evaluate BMI status concerning demographic factors among primary school teachers in the Asir Region, KSA.

**Methods:**

A cross-sectional design was employed, involving 370 primary school teachers. Data were collected using a self-administered questionnaire that covered sociodemographic details, anthropometric measurements, physical activity levels, and nutritional knowledge. Statistical analyses included Pearson’s correlation and Chi-square tests, with significance at p < 0.05.

**Results:**

Among the participants, 38.6% were overweight, and 33.5% were obese, with 76% of married teachers classified as overweight or obese. Only 9.0% exhibited excellent nutritional knowledge, while 25.0% demonstrated poor knowledge. The majority (84.0%) engaged in low or minimal physical activity. A weak but significant correlation was observed between nutritional knowledge and BMI (p < 0.05), whereas no significant associations were found between physical activity levels and either BMI or nutritional knowledge.

**Conclusion:**

The study highlights critical gaps in nutritional knowledge and physical activity among teachers, emphasizing the need for targeted school-based health education programs. Improving teacher health behaviors could enhance their well-being and enable them to act as positive role models for their students.

## Introduction

Primary school teachers represent, particularly, a significant and vital workforce worldwide. Teachers’ health and lifestyle behaviors and their professional performance are highly important. A teacher’s physical and mental health status can directly impact their ability to impart knowledge and foster a conducive learning environment. Moreover, their professional workload and the school environment could affect their health and lifestyle behaviors, including nutritional habits and physical activity [1, 2]. These factors could influence the teachers and the students under their care, as teachers serve as role models, educators, and health promotors [1–3]. High stress and burnout levels due to work have been recognized as factors leading to teacher attrition, underscoring the effect of teacher well-being on the execution of work-related duties [1, 4, 5]. Limited studies have also explored the influence of work-related factors on teachers’ lifestyle behaviors and reported an adverse effect of high workloads on teachers’ participation in healthy lifestyles, ultimately increasing their risk of chronic diseases [6, 7].

Primary school teachers play a pivotal role in socialization and are also critical contributors to enhancing public health. The lifestyle behaviors of these teachers play a crucial role in improving overall wellness, minimizing disease risk, and shaping the health of future generations. They can significantly impact by serving as role models who shun unhealthy eating habits and integrate personal nutritional knowledge into everyday classroom activities [8]. Their interaction and communication skills enable them to serve as a link between schools and healthcare facilities, thereby transferring their knowledge about physical activity and nutrition to their students [9]. It has been observed that teachers’ lifestyles can positively influence students’ health behaviors and overall health [1]. One research indicates that students’ nutritional habits are primarily influenced by their teachers’ nutritional awareness and eating behaviors [10]. However, the easy availability of high-calorie products in school cafeterias poses a challenge to the school community and contributes to poor nutritional status. Therefore, to lower the incidence of overweight and obesity within the school community, it is crucial to encourage healthy eating habits and implement programs to enhance nutritional and physical activity knowledge and practices among this population group [11].

Excessive body weight, including overweight and obesity conditions, poses a significant threat to public health, as well as the economic and social development of countries [12]. In 2022, over 1 billion people globally were living with obesity. Since 1990, obesity rates among adults have more than doubled. Additionally, the data indicated that 43% of adults were classified as overweight in 2022. [13]. A recent comprehensive survey across all regions of KSA revealed that over four years, obesity rates exhibited the highest level recorded at 22.2% in 2020 and the lowest at 21.4% in 2023 [14]. The causes of excessive body weight are multifaceted. They include physiological factors, such as changes in the quantity and distribution of body fat, a sedentary lifestyle, environmental factors, and a diet high in energy density. A lack of sufficient knowledge about healthy eating can also contribute significantly [15]. Poor diet quality, particularly low intake of vegetables, fruits, and whole grains, is a well-established risk factor for chronic diseases and contributes to the global disease burden [16]. Corbett et al. (2024) revealed that 53% of the teachers studied were classified as overweight or obese, 9% met vegetable intake guidelines, and 23% met the recommended physical activity guidelines [6]. Therefore, teachers need support and education through school-based programs. These school-based programs are an ideal environment for optimizing healthy lifestyle behaviors to prevent metabolic-related chronic diseases and promote their well-being to support them in executing their professional duties as health advocates and role models for the students they are responsible for [1, 17, 18]. The health of school teachers is influenced by preventive strategies, including education and awareness about activities that promote health [19]. That is why the Saudi Ministry of Education emphasizes establishing and maintaining a healthy school environment. It also advocates for enhancing health promotion activities in educational settings, especially schools, to improve nutritional awareness and decrease the prevalence of communicable and non-communicable diseases within the school community [20]. However, the Asir Region in southwestern Saudi Arabia faces significant health challenges, particularly concerning obesity among adults due to its growing urbanization and unique, diverse cultural and population traits, mainly resulting in changes in their lifestyle and health behaviors [21]. Despite the crucial role of the teachers in the community as positive role models for students in their healthy dietary intake and lifestyle habits, limited studies have also evaluated the health behaviors of teachers, especially on knowledge levels of nutrition and physical activities, indicating a significant gap in the literature. Furthermore, the limited intervention data suggests teachers’ knowledge could be improved by participating in appropriate educational programs [8, 9, 15, 22, 23]. The correlation between physical activity, BMI, and dietary knowledge has not been studied among primary school teachers in Asir, Saudi Arabia. Therefore, the current study aimed to assess primary school teachers’ nutritional knowledge, physical activity levels, and their relationship with BMI categories in Asir, KSA. Understanding these health behaviors is crucial for setting a baseline for implementing health strategies, policies, and interventions in the school population, including teachers and students.

## Materials and method

### Study design

A cross-sectional design was conducted among male and female primary school teachers actively teaching throughout the 2024 academic year. Each teacher had at least one year of teaching experience in Asir Province, in the southwest region of the Kingdom of Saudi Arabia. The sample size was determined using the Raosoft formula, which represented the total target population of 9629, with a precision level set at 0.05 [24]. The overall sample size calculated was 370, with a 5% margin of error.

A pilot study was conducted to evaluate the questionnaire’s clarity, relevance, and comprehensiveness, ensuring it effectively captured the intended data. This preliminary phase involved 30 teachers selected through simple random sampling. Based on their feedback, adjustments were made to improve question clarity and reduce the survey’s time burden. Notably, participants from the pilot study were excluded from the final analysis. The current study participants were chosen using simple random sampling, with Microsoft Excel’s "RAND" function employed. Approval was obtained from the General Administration of Education of Asir region. The administration then circulates the structured and validated Arabic questionnaires to the selected schoolteachers through official communication channels, such as emails on the Madrasti platform. The questionnaire was adapted by researchers based on a literature review for the current studies [25, 26]. Data collection took place from 02/01/2024 to 18/02/2024.

### Questionnaire

Participants were invited via email to complete a self-administered, 37-item validated, and pretested Arabic language questionnaire created using an online survey system (Google Forms). The questionnaire featured a user-friendly interface and included three sections. The interface outlined the study’s purpose and assured participants of their confidentiality. Responses were collected anonymously, and an ethical statement was included before proceeding to the complete questionnaire; once they agreed on the digital consent form, only the questionnaire would be opened, indicating that completing it would imply informed consent. To promote completeness, specific fields were marked as mandatory.

The first section of the questionnaire focused on gathering general sociodemographic information, lifestyle factors, and anthropometric measurements. This section consisted of ten questions, which included inquiries about age, gender, education, marital status, height, and weight. The chronic morbidity status was also collected. Additionally, this section included smoking status, the type of diet, self-evaluation of the participant’s current nutritional practices, and one question regarding the participants’ self-perception of the level of effect of school health-awareness programs provided by the Education Ministry on their nutritional awareness. The second section evaluated participants’ physical activity levels using the Arabic version of the International Physical Activity Questionnaire-Short Form (IPAQ-SF), a widely used self-report assessment tool with acceptable validity and reliability worldwide. The questionnaire contains seven questions, which asked the participants to report the frequency and duration of their physical activity at different intensity levels (i.e., light, moderate, and vigorous) and their average sedentary time in minutes per day over the past week. The total time (in minutes) spent in vigorous physical activity (VPA) and moderate physical activity (MPA) was calculated by multiplying the frequency (days per week) and duration (minutes/day on average) of each corresponding activity. Additionally, the total time spent (in minutes) in moderate-to-vigorous physical activity (MVPA) was calculated by adding the time spent in MPA and VPA [27]. The third section evaluated the participants’ nutrition knowledge using a modified version of the Consumer Nutrition Knowledge Scale (CoNKS), a concise and effective tool for assessing consumer nutrition knowledge. The modified CoNKS was formulated in English and subsequently translated into Arabic by a qualified translator to ensure clarity and comprehensibility. It included 20 questions that covered declarative and procedural nutrition knowledge, using everyday language instead of scientific terminology. It consisted of three subscales based on content considerations: one for procedural nutrition knowledge (7 items, 0–7 points), one for declarative knowledge of nutrient contents (7 items, 0–7 points), and one for declarative knowledge of calorie content (6 items, 0–6 points). Correct answers were allotted to a value of "1", whereas incorrect answers, "don’t know" responses, were assigned a value of "0". The sum of the 20 items gave the nutrition knowledge score (CoNKS Total), ranging from 0 to 20 points. The content and face validity of CoNKS were rigorously assessed by five expert panels from various fields, including nutrition, community medicine, and public health, through academic email correspondence, and the panel unanimously agreed that it was clear, relevant, and credible. This instrument’s internal consistency was measured using Cronbach’s Alpha (0.591) [28]. Measures included for refining survey items based on expert feedback, conducting a pilot test to ensure clarity, and using complementary analytical methods to validate the findings.

### Statistical methods

The accuracy and completeness of the collected data were thoroughly scrutinized through two rounds of checks. After coding the data, IBM SPSS Statistics for Windows (Version 25.0, IBM Corp., Armonk, NY) was utilized for practical statistical analysis. Descriptive statistics, such as frequency, median, mean, standard deviation, and coefficient of variation, were used to characterize quantitative variables. Descriptive measures, such as means and standard deviations, were employed to analyze continuous dependent and independent variables. Percentages were used to describe categorical data. The association between categorical independent variables and nominal levels was assessed using a Chi-Square test and Pairwise correlation analysis, with significance levels set at 0.05 and 0.01.

### Ethical considerations

King Khalid University’s ethical committee approved this study (Approval No. ECM#2023–903). Informed consent was obtained from all participants in the study through an online questionnaire. A statement informed respondents that their response indicated their informed consent.

## Result

A total of 370 primary school teachers participated in this study; the genders were almost equally distributed: males 181(48.9%) and females 189 (51.1%). Most participants, 241 (65.1%), were aged 30–49, and one-quarter of the participants, 95 (25.7%), were older than 50. Most of the participants, 318 (85.9%), were married. Notably, there was a significant difference in age distribution between genders (p-value < 0.001). Divorced and widowed participants made up smaller proportions. The presence of chronic illness was significantly associated with gender (p-value < 0.001), with a higher percentage of males reporting chronic diseases compared to females. Unhealthy eating behavior was prevalent 222 (60.0%), with a significant difference between genders (p-value = 0.004); healthier eating patterns were more common among females. Non-smokers comprised the majority 311 (84.1%), while current and occasional smokers were relatively rare. Males had a higher prevalence of smoking (p-value < 0.001). The distribution across BMI categories (normal, overweight, obese) did not show significant gender differences (p-value = 0.531). Participants reported varying impacts from health-promoting programs, with high-impact responses more common among females (p-value = 0.001) (Table 1).

**Table 1:** Distribution of study participants based on demographic, clinical profile, and lifestyle pattern (n = 370).

Variables		Gender												p-value
		Female				Male				Total				
		No		%		No		%		No		%		
Age:	<30	29	15.3%		5		2.8%		34		9.2%		0.0001*	
	30–49	139	73.5%		102		56.4%		241		65.1%			
	>50	21	11.1%		74		40.9%		95		25.7%			
Social status	Single	26	13.8%		9		5.0%		35		9.5%		0.003*	
	Married	150	79.4%		168		92.8%		318		85.9%			
	Divorced	9	4.8%		2		1.1%		11		3.0%			
	widow	4	2.1%		2		1.1%		6		1.6%			
Presence of Chronic Illness	No	165	87.3%		131		72.4%		296		80.0%		0.0001*	
	Yes	24	12.7%		50		27.6%		74		20.0%			
Type of eating behaviors	Healthy	91	48.1%		57		31.5%		148		40.0%		0.004*	
	Unhealthy	98	26.5%		124		33.5%		222		60.0%			
Smoking status	Current smoker	3	1.6%		42		23.2%		27		23.3%		0.0001*	
	occasional smoker	2	1.1%		12		6.6%		14		3.8%			
	Ex-smoker	1	0.05%		17		9.4%		18		4.9%			
	Non-smoker	184	97.4%		127		70.2%		311		84.1%			
BMI	Normal	59	15.9%		44		11.8%		103		27.8%		0.531	
	Overweight	70	37.0%		73		40.3%		143		38.6%			
	Obesity	60	31.7%		64		35.4%		124		33.5%			
Self-reported effect of health-promoting programs of the Ministry of Education	No impact	51	27.0%		64		35.4%		115		31.1%		0.001*	
	Low-impact	66	34.9%		81		44.8%		147		39.7%			
	High impact	72	38.1%		36		19.9%		108		29.2%			

χ^2^ test. Statistically significant p-value at ≤ .05, **Statistically significant p-value at ≤ .001.

**Fig 1:**
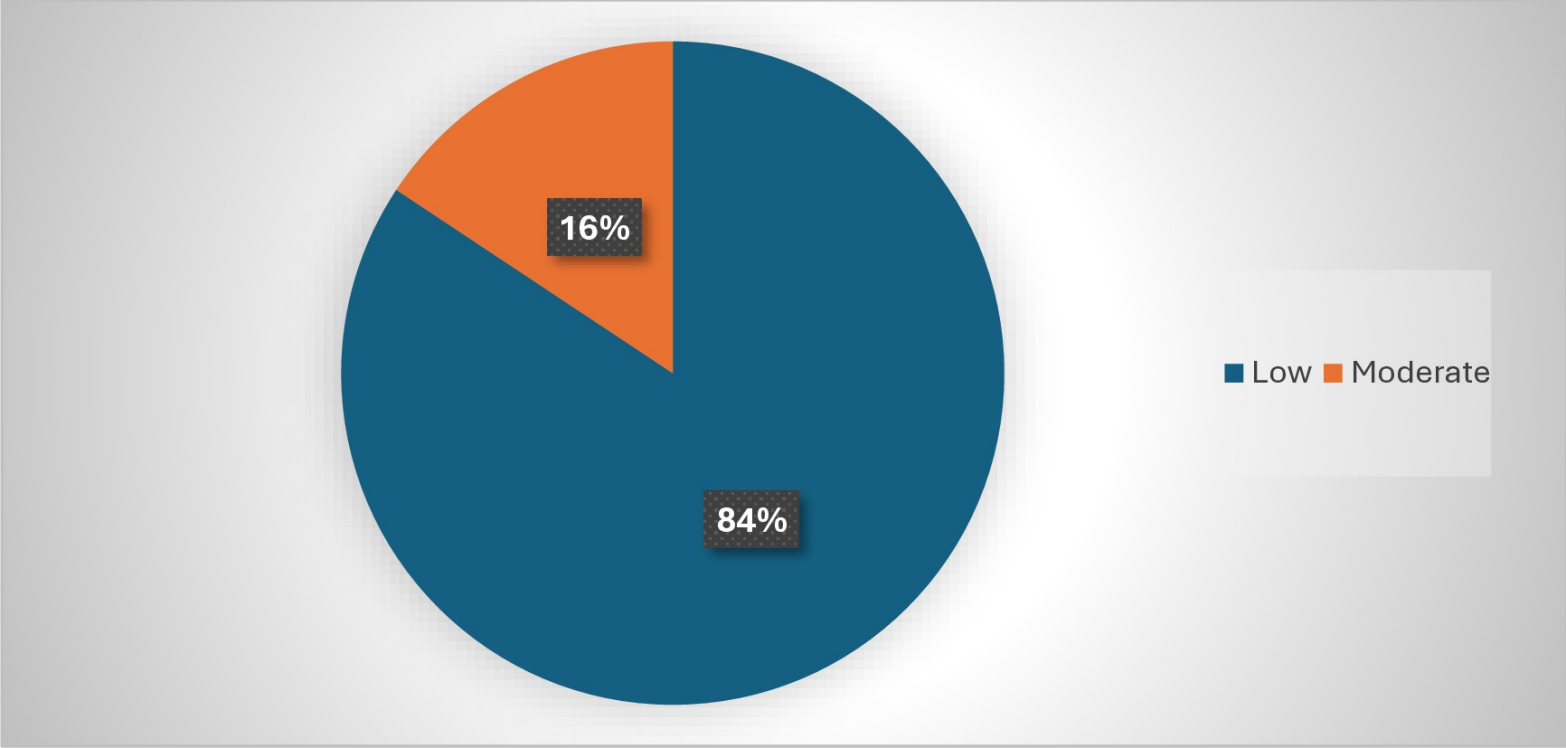
Physical activity levels among the participants.

None of the participants practiced vigorous physical activity (Fig 1). However, the majority (84.0%) reported a low or minimal level of physical activity, and 16.0% reported a moderate level.

**Fig 2:**
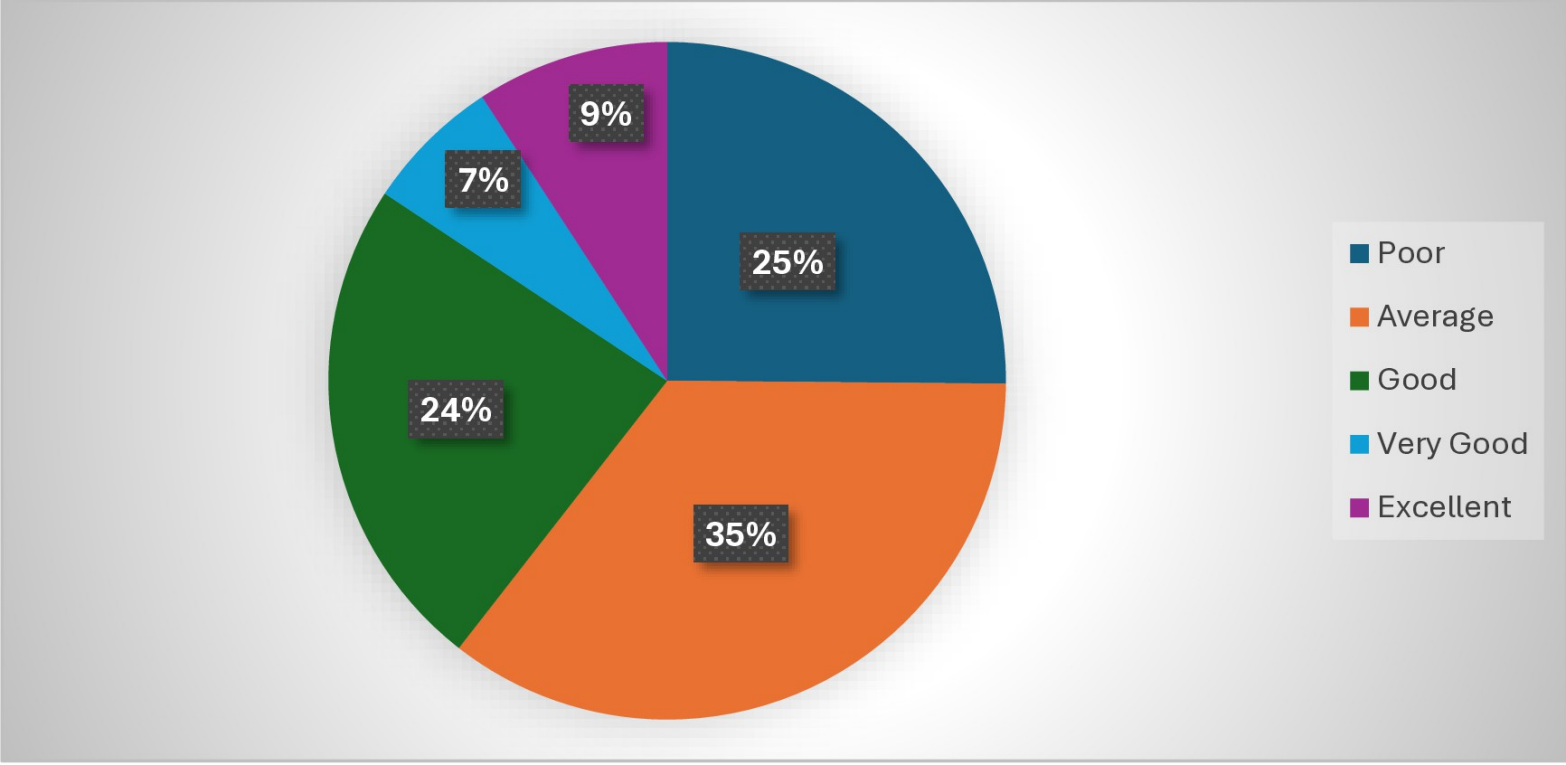
Dietary Knowledge levels among the participants.

Fig 2. underscores nutritional knowledge levels among participants, indicating that 35% possess an average level of nutritional knowledge, while 25.0% are categorized as having poor knowledge. Additionally, only 24.0% have good nutritional knowledge, and 16.0% fall into the very good or excellent categories.

Table 2 reveals that the distribution of BMI categories (normal, overweight, obesity) did not significantly differ between females and males (p = 0.332). However, notable differences were found between age and social status, with a significant association found for both (p = 0.001 and p = 0.000, respectively). Younger participants (under 30) showed a higher proportion of individuals classified as normal weight (55.9%), while those aged 30–49 and over 50 had a greater proportion of individuals classified as overweight or obese. Single participants had a more normal BMI (51.4%) than married, while divorced and widowed had higher percentages of overweight and obesity. Participants following the DASH diet showed a higher proportion of overweight or obesity than other diet categories with statistically significant differences (p = 0.008). Smoking status, physical activity, and nutritional knowledge did not significantly correlate with BMI categories (Table 2).

**Table 2:** Distribution of study subjects based on BMI and demographic profile (n = 370).

Variables		BMI Categories								P value
		Normal		Overweight		Obesity		Total		
		No	%	No	%	No	%	No	%	
Gender	Female	59	31.2%	70	37.0%	60	31.7%	189	100%	0.332
	Male	44	24.3%	73	40.3%	64	35.4%	181	100%	
	Total	103	28%	143	39%	124	34%	370	100%	
Age:	<30	19	55.9%	12	35.3%	3	8.8%	34	100%	.001*
	30–49	65	27.0%	94	39.0%	82	34.0%	241	100%	
	>50	19	20.0%	37	38.9%	39	41.1%	95	100%	
	Total	103	28%	143	39%	124	34%	370	100%	
Social/ Marital status	Single	18	51.4%	16	45.7%	1	2.9%	35	100%	.0001*
	Married	76	23.9%	125	39.3%	117	36.8%	318	100%	
	Divorced	5	45.5%	1	9.1%	5	45.5%	11	100%	
	widow	4	66.7%	1	16.7%	1	16.7%	6	100%	
	Total	103	28%	143	39%	124	34%	370	100%	
Smoking status	current smoker	11	40.7%	8	29.6%	8	29.6%	27	100%	0.542
	Occasional smoker	5	35.7%	7	50.0%	2	14.3%	14	100%	
	Ex-smoker	5	27.8%	7	38.9%	6	33.3%	18	100%	
	Non-smoker	82	26.4%	121	38.9%	108	34.7%	311	100%	
	Total	103	28%	143	39%	124	34%	370	100%	
Type of diet	DASH Diet	0	0.0%	5	45.5%	6	54.5%	11	100%	.008*
	Diabetic diet	7	24.1%	15	51.7%	7	24.1%	29	100%	
	weight control Diet	18	20.7%	35	40.2%	34	39.1%	87	100%	
	low Carb Diet	4	16.0%	7	28.0%	14	56.0%	25	100%	
	No specific Diet	72	33.3%	81	37.5%	63	29.2%	216	100%	
	Vegetarian	2	100.0%	0	0.0%	0	0.0%	2	100%	
	Total	103	28%	143	39%	124	34%	370	100%	
Physical Activity Levels	Low	80	25.6%	123	39.4%	109	34.9%	312	100%	0.084
	Moderate	23	39.7%	20	34.5%	15	25.9%	58	100%	
	Total	103	28%	143	39%	124	34%	370	100%	
Nutritional Knowledge levels	Poor	25	26.9%	37	39.8%	31	33.3%	93	100%	0.309
	Average	37	28.2%	54	41.2%	40	30.5%	131	100%	
	Good	28	31.8%	34	38.6%	26	29.5%	88	100%	
	Very Good	6	25.0%	10	41.7%	8	33.3%	24	100%	
	Excellent	7	20.6%	8	23.5%	19	55.9%	34	100%	
	Total	103	28%	143	39%	124	34%	370	100%	

χ^2^ test.

* Statistically significant p-value at ≤ .05

**Statistically significant p-value at ≤ .001.

The association between nutritional knowledge levels and various demographic, lifestyle, and clinical factors among 370 participants. Male gender exhibited slightly higher representation in the "Excellent" category 18 (9.9%) compared to females 16 (8.5%), the difference was not statistically significant (p = 0.21). Regarding age, participants aged 30–49 demonstrated the highest proportion in the "Average knowledge" category 90 (37.3%), while those above 50 had a greater percentage in the "Excellent knowledge" category 11 (11.6%) compared to younger age groups, though the association was not significant (p = 0.94). Marital status showed interesting trends, with divorced participants predominantly in the "Poor knowledge" category 5 (45.5%), while single individuals had the highest proportion in the "Excellent knowledge" category 5 (14.3%). However, this association was not statistically significant (p = 0.499). Eating habits significantly influenced nutritional knowledge (p = 0.009), as participants with healthy eating habits were more likely to be in the "Excellent knowledge" 20 (13.5%) compared to those with unhealthy habits 9 (6.2%). Dietary patterns also exhibited a significant association (p = 0.001), with specific diets like DASH and low-carb diets correlating with higher nutritional knowledge levels. For instance, 40.0% of low-carb diet followers were in the "Excellent knowledge". Smoking status showed significant differences (p = 0.001), with non-smokers comprising a higher proportion in the "Good knowledge" 68 (21.9%) and "Excellent"25 (8.0%) categories compared to current smokers, none of them were had excellent knowledge. Finally, the presence of chronic illness, while not statistically significant (p = 0.121), revealed that individuals with chronic illnesses had a greater representation in the "Excellent knowledge (16.2%) compared to those without chronic illness 22 (7.4%). (Table 3).

**Table 3:** Association between nutritional knowledge, clinical profile, and lifestyle and demographic pattern among the participants (n = 370).

Variables		Nutritional Knowledge levels										P- Value
		Poor		Average		Good		Very Good		Excellent		
		No	%	No	%	No	%	No	%	No	%	
Gender	Female	39	20.6%	75	39.7%	45	23.8%	14	7.4%	16	8.5%	0.21
	Male	54	29.8%	56	30.9%	43	23.8%	10	5.5%	18	9.9%	
	Total	93	25.1%	131	35.4%	88	23.8%	24	6.5%	34	9.2%	
Age	<30	9	26.5%	12	35.3%	7	20.6%	3	8.8%	3	8.8%	0.94
	30–49	61	25.3%	90	37.3%	56	23.2%	14	5.8%	20	8.3%	
	>50	23	24.2%	29	30.5%	25	26.3%	7	7.4%	11	11.6%	
	Total	93	25.1%	131	35.4%	88	23.8%	24	6.5%	34	9.2%	
Social/ Marital status	Single	5	14.3%	13	37.1%	10	28.6%	2	5.7%	5	14.3%	0.499
	Married	82	29.1%	112	39.7%	37	13.1%	22	7.8%	29	10.3%	
	Divorced	5	45.5%	2	18.2%	4	36.4%	0	0.0%	0	0.0%	
	widow	1	16.7%	4	66.7%	1	16.7%	0	0.0%	0	0.0%	
	Total	93	25.1%	131	35.4%	88	23.8%	24	6.5%	34	9.2%	
Type of eating habits/ behavior	Healthy	42	28.4%	39	26.4%	38	25.7%	9	6.1%	20	13.5%	0.009*
	Unhealthy	39	26.7%	64	43.8%	26	17.8%	8	5.5%	9	6.2%	
	I don’t know	12	15.8%	28	36.8%	24	31.6%	7	9.2%	5	6.6%	
	Total	93	25.1%	131	35.4%	88	23.8%	24	6.5%	34	9.2%	
Type of diet	DASH Diet	3	27.3%	0	0.0%	1	9.1%	5	45.5%	2	18.2%	0.001*
	Diabetic diet	2	6.9%	15	51.7%	10	34.5%	0	0.0%	2	6.9%	
	Weight control Diet	20	23.0%	30	34.5%	26	29.9%	3	3.4%	8	9.2%	
	Low Carb Diet	8	32.0%	5	20.0%	2	8.0%	0	0.0%	10	40.0%	
	Vegetarian diet	0	0.0%	0	0.0%	2	100.0%	0	0.0%	0	0.0%	
	No specific diet	60	27.8%	81	37.5%	47	21.8%	16	7.4%	12	5.6%	
	Total	93	25.1%	131	35.4%	88	23.8%	24	6.5%	34	9.2%	
Smoking status	Current smoker	0	0.0%	13	48.1%	13	48.1%	1	3.7%	0	0.0%	0.001*
	Occasional smoker	7	50.0%	3	21.4%	2	14.3%	0	0.0%	2	14.3%	
	Ex-smoker	2	11.1%	2	11.1%	5	27.8%	2	11.1%	7	38.9%	
	Non-smoker	84	27.0%	113	36.3%	68	21.9%	21	6.8%	25	8.0%	
	Total	93	25.1%	131	35.4%	88	23.8%	24	6.5%	34	9.2%	
Presence of chronic illness	No	80	27.0%	106	35.8%	69	23.3%	19	6.4%	22	7.4%	0.121
	Yes	13	17.6%	25	33.8%	19	25.7%	5	6.8%	12	16.2%	
	Total	93	25.1%	131	35.4%	88	23.8%	24	6.5%	34	9.2%	

χ^2^ test

* Statistically significant p-value at ≤ .05

**Statistically significant p-value at ≤ .001.

Table 4 explores the relationship between physical activity levels (low and moderate) and demographic, lifestyle, and clinical factors among 370 participants. The gender, both males 160 (83.98%) and females 152 (84.66%) predominantly reported low physical activity, with no significant difference observed between the groups (p = 0.858). Age under 30 years were more likely to engage in moderate physical activity 12 (35.3%) compared to those aged 30–49 years 26 (11.0%) and above 50 years 20 (21.0%) the difference showed a significant association with physical activity levels (p = 0.001). Marital status single participants reported slightly higher levels of moderate physical activity 10 (29.0%) compared to married individuals 45 (14.0%) although it did not show significant differences (p = 0.17). Eating habits were significantly associated with physical activity levels (p = 0.05), with participants reporting healthy eating habits more likely to engage in moderate physical activity 30 (20.0%) compared to those with unhealthy habits 22 (15.0%) or those unsure of their eating habits 6 (8.0%). Dietary patterns also exhibited a significant association (p = 0.05). The participants on weight control diets and low-carb diets showed higher levels of moderate physical activity 21 (24.0% each) compared to those with no specific diet 24 (11.0%). Smoking status, however, did not significantly influence physical activity levels (p = 0.949), the current smokers reported a higher proportion of moderate physical activity 4 (57.0%) compared to non-smokers 50 (16.0%). Coming to the presence of chronic illness was not significantly associated with physical activity levels (p = 0.886), with both participants with 12 (16.0%) and without 12 (16.0%) chronic illness reporting similar distributions in moderate physical activity (Table 4).

**Table 4:** Association between physical activity and clinical profile, lifestyle, and demographic pattern among the participants (n = 370).

Variables	Physical activity grade							
		Low		Moderate		Total		P- Value
		No	%	No	%			
Gender	Female	160	84.7%	29	15.3%	189	100%	0.858
	Male	152	84.0%	29	16.0%	181	100%	
	Total	312	84.3%	58	15.7%	370	100%	
Age	<30	22	64.7%	12	35.3%	34	100%	0.001*
	30–49	215	89.2%	26	10.8%	241	100%	
	>50	75	78.9%	20	21.1%	95	100%	
	Total	312	84.3%	58	15.7%	370	100%	
Social/ Marital status	Single	25	71.4%	10	28.6%	35	100%	0.17
	Married	273	85.8%	45	14.2%	318	100%	
	Divorced	9	81.8%	2	18.2%	11	100%	
	widow	5	83.3%	1	16.7%	6	100%	
	Total	312	84.3%	58	15.7%	370	100%	
Type of eating habits/ behavior	Healthy	118	79.7%	30	20.3%	148	100%	0.05*
	Unhealthy	124	84.9%	22	15.1%	146	100%	
	I don’t know	70	92.1%	6	7.9%	76	100%	
	Total	312	84.3%	58	15.7%	370	100%	
Type of diet	DASH Diet	10	90.9%	1	9.1%	11	100%	0.05*
	Diabetic diet	23	79.3%	6	20.7%	29	100%	
	Weight control Diet	66	75.9%	21	24.1%	87	100%	
	Low Carb Diet	19	76.0%	6	24.0%	25	100%	
	Vegetarian diet	2	100.0%	0	0.0%	2	100%	
	No specific diet	192	88.9%	24	11.1%	216	100%	
	Total	312	84.3%	58	15.7%	370	100%	
Smoking status	Current smoker	3	42.9%	4	57.1%	7	100%	0.949
	Occasional smoker	12	85.7%	2	14.3%	14	100%	
	Ex-smoker	16	88.9%	2	11.1%	18	100%	
	Non-smoker	261	83.9%	50	16.1%	311	100%	
	Total	312	84.3%	58	15.7%	370	100%	
Presence of chronic illness	No	250	84.5%	46	15.5%	296	100%	0.886
	Yes	62	83.8%	12	16.2%	74	100%	
	Total	312	84.3%	58	15.7%	370	100%	

* Statistically significant p-value at ≤ .05

**Statistically significant p-value at ≤ .001.

Table 5 shows the correlation between the participants’ physical activity and nutritional knowledge scores, which were weak and insignificant. The correlation between physical activity score and body mass index was an indirectly weak, insignificant correlation. However, the correlation between nutritional knowledge score and BMI categories was found to be a direct weak correlation with statistically significant (p<0.05) (Table 5).

**Table 5:** Correlation among BMI, nutritional knowledge, and physical activity of the participants (n = 370).

Correlation				
Spearman’s rho		Physical Activity Overall Scores	Nutritional Knowledge Overall Scores	BMI
Physical Activity Overall Scores	Correlation Coefficient	1.000	0.027	-0.101
	Sig. (2-tailed)		0.608	0.053
	N	370	370	370
Nutritional Knowledge Overall Scores	Correlation Coefficient	0.027	1.000	0.123*
	Sig. (2-tailed)	0.608		0.018*
	N	370	370	370
BMI	Correlation Coefficient	-0.101	0.123*	1.000
	Sig. (2-tailed)	0.053	0.018*	
	N	370	370	370

*Correlation is significant at the 0.05 level (2-tailed)

## Discussion

This study aimed to assess primary school teachers’ physical activity and nutritional knowledge levels in the Asir Region, Saudi Arabia, focusing on understanding the factors associated with these health behaviors. The findings highlight several critical issues regarding teachers’ health status and lifestyle behaviors, significantly affecting their role as educators and health promoters within the school community.

The results revealed that a substantial proportion of the teachers were classified as either overweight or obese, with nearly 72.1% of the participants in these categories. This aligns with previous studies conducted in Saudi Arabia and other regions, which have reported high rates of overweight and obesity among teachers, particularly in middle-aged populations [29]. Another study in Jeddah by Bakhotmah et al. observed 40% of Obesity among school teachers [30]. The high prevalence of overweight and obesity among teachers raises concerns about their health and their ability to serve as role models for healthy living, given the well-established link between excessive body weight and various chronic diseases, including cardiovascular diseases, type 2 diabetes, and hypertension. Studies conducted by Delfino et al. and Korkmaz C. et al. also observed that Obesity is associated with the Health Behaviors of Teachers and comorbidities among schoolteachers [31, 32]. The high rates of overweight and obesity among teachers suggest a dual challenge. On the one hand, their health may impede their ability to fulfill their responsibilities effectively, potentially affecting their productivity and well-being. On the other hand, as role models, their health behaviours directly influence students’ perceptions of healthy lifestyles. If teachers are to champion health education effectively, they must embody the principles of balanced nutrition and active living. These findings emphasize the need to integrate teacher-focused health promotion programs into school settings. Addressing obesity and sedentary lifestyles requires personalized strategies, including accessible physical activity programs, stress management training, and policies that encourage healthier eating habits at school.

The study reveals that a significant proportion of the participants engaged in low or minimal physical activity, with none reporting vigorous physical activity and only 16% engaging in moderate physical activity. This finding is concerning, considering the critical role of physical activity in maintaining a healthy weight and preventing chronic diseases. The low levels of physical activity observed in this study are consistent with global trends of sedentary lifestyles, particularly among professionals with demanding workloads, such as teachers. The lack of physical activity among the teachers in this study could be attributed to their challenges in balancing their professional responsibilities with personal health practices. Similar studies also observed that most schoolteachers, regardless of their role in the classroom, are moderately physically active during teaching.[33, 34]. Among the public, including schoolteachers, with a degree of physical inactivity, sedentary life, and nutritional behavior, the risk of obesity increases. Obesity is connected with lifestyle and comorbidities among schoolteachers. [31]. The lack of vigorous physical activity among participants highlights the systemic challenges faced by professionals in balancing work and personal health. Encouragingly, moderate physical activity levels, though limited, indicate a foundation upon which schools and policymakers can build. School-based health promotion programs offering structured physical activity opportunities for teachers, such as fitness classes or active breaks, could significantly enhance their physical activity levels. Additionally, collaboration with community resources like fitness centers or sports clubs may provide cost-effective solutions to support teachers’ health.

Nutritional knowledge among the primary school teachers was low, with only 9.0% of participants demonstrating excellent knowledge of nutritional issues [35]. There was a significant association between higher levels of nutritional knowledge and better nutritional practices, as those with excellent or good expertise were more likely to practice healthy eating habits [36]. On the other hand, those with poor nutritional knowledge were less likely to follow a healthy diet, which could exacerbate the risk of obesity and related chronic conditions. This finding is particularly concerning, given that teachers are expected to influence their students’ eating behaviors positively [37]. Teachers’ nutritional knowledge plays a crucial role in imparting knowledge to the students and facilitating greater acceptance of nutrition education by the children when taught by teachers rather than information provided by an outside nutrition expert [38]. The low levels of nutritional knowledge observed among the teachers in this study highlight a critical gap that needs to be addressed through professional development programs focusing on nutrition education [39]. Nutritional knowledge emerged as a critical determinant of healthy eating behaviours. However, the study identified substantial gaps in teachers’ understanding of nutrition, underscoring the need for comprehensive, ongoing professional development programs. Schools can incorporate nutrition workshops into teacher training curricula, addressing practical strategies for implementing balanced diets and teaching these principles to students. By enhancing teachers’ knowledge, schools not only empower them to make healthier choices but also strengthen their ability to influence their students’ dietary habits positively.

The study highlighted that teachers with chronic illnesses had better nutritional knowledge than those without such conditions. This suggests that the experience of managing a chronic disease may drive individuals to seek more information about healthy eating, either through physician advice or personal research [40]. However, this reactive approach to acquiring nutritional knowledge underscores the importance of proactive health education for all teachers, regardless of their current health status, to prevent the onset of chronic diseases. A higher proportion of overweight or obesity was observed in participants following the DASH diet. One possible explanation for this is the lack of explicit calorie restriction. While the DASH diet emphasizes consuming nutrient-dense foods such as fruits, vegetables, and whole grains, it does not specifically limit caloric intake. Participants may have increased their portions or consumed more calorie-dense foods, leading to a potential rise in overall caloric consumption and subsequent weight gain. Variations in adherence, differences in physical activity levels, or individual metabolic responses to the diet could further influence weight outcomes. The weak correlations observed between physical activity scores, nutritional knowledge scores, and BMI suggest that while knowledge is necessary, it is insufficient to drive behavior change [41]. Despite some participants’ good nutritional knowledge, this did not always translate into healthy lifestyle practices like regular physical activity or maintaining a healthy weight.[42]. This disconnect between knowledge and behavior may be due to various factors, including high-stress levels, lack of time, and environmental barriers, such as the availability of unhealthy food options at school cafeterias [43]. These findings emphasize the need for a more holistic approach to health promotion among teachers, which provides knowledge and addresses the practical and environmental challenges that may hinder healthy behavior.

The main limitation of this study is its cross-sectional design; hence, it cannot establish the causality of the variables. While the study offers valuable insights into the current levels of nutritional knowledge and physical activity among primary school teachers, it does not consider temporal changes or factors influencing these behaviors over time. Additionally, the reliance on self-reported data may introduce bias, as participants might underreport or overreport their physical activity levels and nutritional knowledge. Although the sampling method was randomized, it may not fully capture the diversity of experiences among the entire population of teachers, potentially attenuating the generalizability of the findings.

### Recommendations

Schools should develop and implement structured wellness programs for teachers, promoting both physical activity and nutritional education. These programs could include regular health check-ups, fitness challenges, and on-site access to nutritionists or dieticians. Policymakers can be involved in creating active school environments, providing healthier cafeteria options, and offering incentives for teachers who participate in health promotion activities. Training teachers as health ambassadors within their schools can multiply the impact of health promotion efforts. Addressing barriers such as the availability of unhealthy food options and the lack of opportunities for physical activity within schools is crucial. Partnerships with local businesses to provide healthy meals and snacks in cafeterias could be explored. Similarly, adapting school infrastructure to facilitate physical activities for teachers during breaks could foster a more active culture. Future research should adopt longitudinal designs to explore the causal relationships between teachers’ health behaviors, knowledge, and their roles as educators. Exploring the interplay of psychosocial factors, such as stress, work-life balance, and cultural attitudes toward health, could provide deeper insights into teachers’ barriers to adopting healthier lifestyles.

## Conclusion

Primary school teachers’ health challenges are critical for fostering a healthier and more productive educational environment. Schools and policymakers can create a ripple effect by prioritizing teacher wellness through targeted interventions and promoting healthier lifestyles for teachers, their students, and the wider community.

## References

[1] Jakstas T, Follong B, Bucher T, Miller A, Shrewsbury VA, Collins CE. Addressing schoolteacher food and nutrition-related health and wellbeing: a scoping review of the food and nutrition constructs used across current research. International Journal of Behavioral Nutrition and Physical Activity. 2023;20(1):108. doi: 10.1186/s12966-023-01502-5 37700281 PMC10498614

[2] Hamilton L, Goodman L, Roberts L, Dial LA, Pratt M, Musher-Eizenman D. Teacher Experience, Personal Health, and Dieting Status Is Associated With Classroom Health-Related Practices and Modeling. J Sch Health. 2020. Epub 20201202. doi: 10.1111/josh.12985 . 33289085

[3] Byrne J, Rietdijk W, Pickett K. Teachers as health promoters: Factors that influence early career teachers to engage with health and wellbeing education. Teaching and Teacher Education. 2018;69:289–99. doi: 10.1016/j.tate.2017.10.020

[4] Vo DT, Allen K-A. A systematic review of school-based positive psychology interventions to foster teacher wellbeing. Teachers and Teaching. 2022;28(8):964–99. doi: 10.1080/13540602.2022.2137138

[5] Carroll A, Forrest K, Sanders-O’Connor E, Flynn L, Bower JM, Fynes-Clinton S, et al. Teacher stress and burnout in Australia: examining the role of intrapersonal and environmental factors. Soc Psychol Educ. 2022;25(2):441–69. doi: 10.1007/s11218-022-09686-7 35233183 PMC8874312

[6] Corbett L, Phongsavan P, Okely AD, Peralta LR, Bauman A. A cross-sectional study of Australian teachers’ health: are work-related factors associated with lifestyle behaviours? Health Promot Int. 2024;39(1). doi: 10.1093/heapro/daad192 ; PubMed Central PMCID: PMC10781439. 38198724 PMC10781439

[7] Dias DF, Loch MR, González AD, Andrade SM, Mesas AE. Insufficient free-time physical activity and occupational factors in Brazilian public school teachers. Rev Saude Publica. 2017;51:68. Epub 20170720. doi: 10.1590/S1518-8787.2017051006217 ; PubMed Central PMCID: PMC5510795. 28746571 PMC5510795

[8] Pekğöz A, Şekercioğlu M. Determination of the Nutritional Habits of the Primary Teacher Candidates for the Game and Physical Activities Teaching Course. Journal of Educational Issues. 2022;8:557. doi: 10.5296/jei.v8i1.19826

[9] Tilles-Tirkkonen T, Nuutinen O, Sinikallio S, Poutanen K, Karhunen L. Theory-informed nutrition education curriculum Tools For Feeling Good promotes healthy eating patterns among fifth grade pupils: cross-sectional study. J Hum Nutr Diet. 2018;31(5):647–57. Epub 20180610. doi: 10.1111/jhn.12568 . 29888471

[10] Elmas C, Arslan P. Effect of nutrition education received by teachers on primary school students’ nutrition knowledge. Nutr Res Pract. 2020;14:532. doi: 10.4162/nrp.2020.14.5.532 33029292 PMC7520566

[11] Burrows TL, Whatnall MC, Patterson AJ, Hutchesson MJ. Associations between Dietary Intake and Academic Achievement in College Students: A Systematic Review. Healthcare (Basel). 2017;5(4). Epub 20170925. doi: 10.3390/healthcare5040060 ; PubMed Central PMCID: PMC5746694. 28946663 PMC5746694

[12] Goettler A, Anna G, Sonntag D. Productivity loss due to overweight and obesity: A systematic review of indirect costs. BMJ Open. 2017;7. doi: 10.1136/bmjopen-2016-014632 28982806 PMC5640019

[13] Phelps NH, Singleton RK, Zhou B, Heap RA, Mishra A, Bennett JE, et al. Worldwide trends in underweight and obesity from 1990 to 2022: a pooled analysis of 3663 population-representative studies with 222 million children, adolescents, and adults. The Lancet. 2024;403(10431):1027–50. doi: 10.1016/S0140-6736(23)02750-2 38432237 PMC7615769

[14] Althumiri NA, Bindhim NF, Al-Rayes SA, Alumran A. Mapping Obesity Trends in Saudi Arabia: A Four-Year Description Study. Healthcare. 2024;12(20):2092. doi: 10.3390/healthcare12202092 39451507 PMC11507986

[15] Saintila J, Calizaya-Milla YE, Calizaya-Milla SE, Elejabo-Pacheco AA, Sandoval-Valentin GA, Rodriguez-Panta SG. Association Between Nutritional Knowledge, Dietary Regimen, and Excess Body Weight in Primary School Teachers. J Multidiscip Healthc. 2022;15:2331–9. Epub 20221014. doi: 10.2147/JMDH.S385713 ; PubMed Central PMCID: PMC9578462. 36267850 PMC9578462

[16] Afshin A, Sur PJ, Fay KA, Cornaby L, Ferrara G, Salama JS, et al. Health effects of dietary risks in 195 countries, 1990–2017: a systematic analysis for the Global Burden of Disease Study 2017. The Lancet. 2019;393(10184):1958–72. doi: 10.1016/S0140-6736(19)30041-8 30954305 PMC6899507

[17] Althumiri NA, Basyouni MH, AlMousa N, AlJuwaysim MF, Almubark RA, BinDhim NF, et al. Obesity in Saudi Arabia in 2020: Prevalence, Distribution, and Its Current Association with Various Health Conditions. Healthcare (Basel). 2021;9(3). Epub 20210311. doi: 10.3390/healthcare9030311 ; PubMed Central PMCID: PMC7999834. 33799725 PMC7999834

[18] Dreer-Goethe B, Gouase N. Interventions fostering well-being of schoolteachers: a review of research. Oxford Review of Education. 2021;48. doi: 10.1080/03054985.2021.2002290

[19] Bentsen P, Mygind L, Elsborg P, Nielsen G, Mygind E. Education outside the classroom as upstream school health promotion: ‘adding-in’ physical activity into children’s everyday life and settings. Scand J Public Health. 2022;50(3):303–11. doi: 10.1177/1403494821993715 . 33624553

[20] Al-Shewear N, Bawazir A. Assessment of School Health in Saudi Arabia: The Path to Improved Future Students Health. (Implications of the Saudi School Health Program) Citation: Nora Abdulrhman Al-Shewear, Amen A Bawazir (2022) Assessment of School Health in Saudi Arabia: The Path to Improved Future Students Health. (Implications of the Saudi School Health Program). 2022;5:1.

[21] Al-Shahrani AM, Alqahtani SM, Alghamdi MA, Albalhsn H, Almalhan LA, Alamri MM, et al. Awareness of Obesity and Weight Loss Management Among Adults in the Asir Region, Saudi Arabia. Cureus. 2024;16(12):e75066. doi: 10.7759/cureus.75066 39759738 PMC11695645

[22] Katsagoni CN, Apostolou A, Georgoulis M, Psarra G, Bathrellou E, Filippou C, et al. Schoolteachers’ Nutrition Knowledge, Beliefs, and Attitudes Before and After an E-Learning Program. J Nutr Educ Behav. 2019;51(9):1088–98. Epub 20190808. doi: 10.1016/j.jneb.2019.07.001 . 31402288

[23] Habib-Mourad C, Ghandour LA, Maliha C, Awada N, Dagher M, Hwalla N. Impact of a one-year school-based teacher-implemented nutrition and physical activity intervention: main findings and future recommendations. BMC Public Health. 2020;20(1):256. doi: 10.1186/s12889-020-8351-3 32075607 PMC7031897

[24] Raosoft. Raosoft Sample Size Calculator Seattle: Raosoft, Inc; 2004 [cited 2024]. Available from: http://www.raosoft.com/samplesize.html.

[25] Bany-Yasin H, Elmor AA, Ebrahim BK, Ahmed AAM, Alarachi MR, Abedalqader L, et al. Exploration of the nutrition knowledge among general population: multi-national study in Arab countries. BMC Public Health. 2023;23(1):1178. Epub 20230619. doi: 10.1186/s12889-023-15791-9 ; PubMed Central PMCID: PMC10278315. 37337137 PMC10278315

[26] Koch F, Hoffmann I, Claupein E. Types of Nutrition Knowledge, Their Socio-Demographic Determinants and Their Association With Food Consumption: Results of the NEMONIT Study. Front Nutr. 2021;8:630014. Epub 20210212. doi: 10.3389/fnut.2021.630014 ; PubMed Central PMCID: PMC7907003. 33644108 PMC7907003

[27] Craig CL, Marshall AL, Sjöström M, Bauman AE, Booth ML, Ainsworth BE, et al. International physical activity questionnaire: 12-country reliability and validity. Med Sci Sports Exerc. 2003;35(8):1381–95. doi: 10.1249/01.MSS.0000078924.61453.FB . 12900694

[28] Dickson-Spillmann M, Siegrist M, Keller C. Development and validation of a short, consumer-oriented nutrition knowledge questionnaire. Appetite. 2011;56(3):617–20. Epub 20110215. doi: 10.1016/j.appet.2011.01.034 . 21310201

[29] Almoraie NM, Shatwan IM, Althaiban MA, Hanbazaza MA, Wazzan HA, Aljefree NM. Associations between dietary intake, physical activity, and obesity among public school teachers in Jeddah, Saudi Arabia. Front Nutr. 2023;10:1081928. Epub 20230124. doi: 10.3389/fnut.2023.1081928 ; PubMed Central PMCID: PMC9902718. 36761223 PMC9902718

[30] Bakhotmah B. Teachers Dietary Practices during School Day in Jeddah, Western Saudi Arabia. Food and Nutrition Sciences. 2012;03:1553–60. doi: 10.4236/fns.2012.311203

[31] Delfino LD, Tebar WR, Tebar F, JM DES, Romanzini M, Fernandes RA, et al. Association between sedentary behavior, obesity and hypertension in public school teachers. Ind Health. 2020;58(4):345–53. Epub 20200131. doi: 10.2486/indhealth.2019-0170 ; PubMed Central PMCID: PMC7417500. 32009026 PMC7417500

[32] Korkmaz C. Assessment of Health-Promoting Lifestyle Profiles and Nutritional Knowledge Levels of Pre-Service Physical Education and Science Teachers: A Comparative Study Example 2022.

[33] Kruger HS, Venter CS, Vorster HH, Margetts BM. Physical inactivity is the major determinant of obesity in black women in the North West Province, South Africa: the THUSA study. Transition and Health During Urbanisation of South Africa. Nutrition. 2002;18(5):422–7. doi: 10.1016/s0899-9007(01)00751-1 . 11985949

[34] Brito WF, Santos CL, Marcolongo Ado A, Campos MD, Bocalini DS, Antonio EL, et al. Physical activity levels in public school teachers. Rev Saude Publica. 2012;46(1):104–9. doi: 10.1590/s0034-89102012000100013 . 22249754

[35] Hamulka J, Wadolowska L, Hoffmann M, Kowalkowska J, Gutkowska K. Effect of an Education Program on Nutrition Knowledge, Attitudes toward Nutrition, Diet Quality, Lifestyle, and Body Composition in Polish Teenagers. The ABC of Healthy Eating Project: Design, Protocol, and Methodology. Nutrients. 2018;10(10). Epub 20181005. doi: 10.3390/nu10101439 ; PubMed Central PMCID: PMC6213798. 30720795 PMC6213798

[36] Carbonneau E, Lamarche B, Provencher V, Desroches S, Robitaille J, Vohl MC, et al. Associations Between Nutrition Knowledge and Overall Diet Quality: The Moderating Role of Sociodemographic Characteristics-Results From the PREDISE Study. Am J Health Promot. 2021;35(1):38–47. Epub 20200609. doi: 10.1177/0890117120928877 . 32515200

[37] Parker EA, Feinberg TM, Lane HG, Deitch R, Zemanick A, Saksvig BI, et al. Diet quality of elementary and middle school teachers is associated with healthier nutrition-related classroom practices. Prev Med Rep. 2020;18:101087. Epub 20200402. doi: 10.1016/j.pmedr.2020.101087 ; PubMed Central PMCID: PMC7155219. 32309116 PMC7155219

[38] Cotton W, Dudley D, Peralta L, Werkhoven T. The effect of teacher-delivered nutrition education programs on elementary-aged students: An updated systematic review and meta-analysis. Prev Med Rep. 2020;20:101178. Epub 20200813. doi: 10.1016/j.pmedr.2020.101178 ; PubMed Central PMCID: PMC7481566. 32944494 PMC7481566

[39] Kana’’An H, Saadeh R, Zruqait A, Alenezi M. Knowledge, attitude, and practice of healthy eating among public school teachers in Kuwait. J Public Health Res. 2021;11(2). Epub 20210723. doi: 10.4081/jphr.2021.2223 ; PubMed Central PMCID: PMC8941308. 34313090 PMC8941308

[40] Willett WC KJ, Nugent R, et al. Prevention of Chronic Disease using Diet and Lifestyle Changes. Jamison DT BJ, Measham AR, et al., editor. Washington (DC): The International Bank for Reconstruction and Development / The World Bank; 2006. 21250366

[41] Akpene Amenya PC, Annan RA, Apprey C, Agbley EN. The relationship between nutrition and physical activity knowledge and body mass index-for-age of school-aged children in selected schools in Ghana. Heliyon. 2021;7(11):e08298. Epub 20211101. doi: 10.1016/j.heliyon.2021.e08298 ; PubMed Central PMCID: PMC8577141. 34778586 PMC8577141

[42] Alzaben AS, Aljahdali AA, Alasousi LF, Alzaben G, Kennedy L, Alhashem A. Nutritional Knowledge, Attitudes, and Practices among Family Physician Practitioners in Gulf Countries (Bahrain, Kuwait, Saudi Arabia, and UAE). Healthcare (Basel). 2023;11(19). Epub 20230927. doi: 10.3390/healthcare11192633 ; PubMed Central PMCID: PMC10572505. 37830670 PMC10572505

[43] Warber JI, Warber JP, Simone KA. Assessment of general nutrition knowledge of nurse practitioners in New England. J Am Diet Assoc. 2000;100(3):368–70. doi: 10.1016/S0002-8223(00)00112-7 . 10719416

